# The Role of Alpha Blockers in Children with Dysfunctional Voiding

**DOI:** 10.1100/tsw.2009.98

**Published:** 2009-09-01

**Authors:** Paul F. Austin

**Affiliations:** Pediatric Urology, St. Louis Children's Hospital, Washington University School of Medicine, St. Louis, MO, USA

**Keywords:** adrenergic alpha-antagonists, urination disorders, child

## Abstract

Incomplete bladder emptying or dysfunctional voiding is a common lower urinary tract dysfunction encountered in children. Alpha blocker therapy is used to facilitate bladder emptying in the adult population and has likewise been applied to the pediatric population. Alpha blocker therapy seems well tolerated in children and appears efficacious towards improving bladder emptying in a variety of pediatric voiding disorders. Long-term follow-up and further investigation are warranted in order to validate the role of alpha blocker therapy in pediatric dysfunctional voiding.

## LOWER URINARY TRACT DYSFUNCTION

Functional disturbances of the lower urinary tract (LUT) in children are commonly encountered in pediatric urology and affect many children. They are generally not a serious problem, but are certainly a nuisance. LUT dysfunction involves a spectrum of disorders that present with a variety of symptoms. In order to diminish confusion on the evaluation of children with LUT dysfunction, the International Children's Continence Society (ICCS) has provided new definitions and a standardized terminology to describe and categorize pediatric LUT function and dysfunction[1]. The ICCS categorizes LUT dysfunction with respect to symptoms that are classified according to their relation to the voiding and/or storage phase of bladder function. Distinguishing LUT abnormalities that occur during voiding as opposed to storage is important because these different phases of bladder function lend themselves to different treatment modalities.

## DYSFUNCTIONAL VOIDING: DIAGNOSIS AND TREATMENT

LUT dysfunction that occurs during the voiding phase has been termed "dysfunctional voiding", and does not apply to the storage or filling aspect of the LUT. The ICCS defines dysfunctional voiding as when a neurologically normal child habitually contracts the urethral sphincter during voiding, as observed by uroflow measurements[1]. Tools used to identify dysfunctional voiding include a detailed elimination history, bladder diary, urine flow measurement with uroflowmetry, and evaluation of postvoid residual urine using an ultrasound. A heterogeneous display of signs and symptoms are seen stemming from incomplete emptying and include incontinence, urinary tract infections (UTIs), and irritative voiding symptoms (e.g., frequency, urgency, and dysuria). Incomplete bladder emptying is also characterized by elevated postvoid residual (PVR) using ultrasound, and display of a "staccato" or prolonged urine flow pattern during uroflowmetry.

The initial approach for the majority of children with dysfunctional voiding is behavioral modification and this treatment includes timed voiding, double voiding, relaxation techniques, and treatment of any pertinent bowel elimination issues. When behavioral modification is unsuccessful in the treatment of children with dysfunctional voiding, ancillary measures are then necessary. These other treatment modalities include pelvic floor rehabilitation (also known as urotherapy or biofeedback) and pharmacologic therapy. The objective of this paper is to present and discuss the pharmacologic approach in the treatment of children with dysfunctional voiding.

## LUT PHARMACOLOGIC TARGETS

There are two available targets for pharmacologic therapy in children with dysfunctional voiding. These include the bladder body (specifically the detrusor muscle) and the bladder outlet (specifically the bladder neck and proximal urethra). Pharmacologic agents that target the detrusor muscle include bladder relaxants and anticholinergic therapies that facilitate storage by relaxing the detrusor smooth muscle. Unfortunately, there are no studies that show evidence that cholinergic agonists (e.g., bethanechol) can be used to facilitate bladder emptying significantly. Subsequently, pharmacologic measures have focused on promoting bladder emptying by targeting the bladder outlet using alpha-adrenergic antagonists ("alpha blockers").

## ALPHA-ADRENERGIC RECEPTOR SITES

Alpha-adrenergic receptors have been demonstrated in the LUT, with a large concentration located at the bladder neck and throughout the human urethra[2,3] (Fig. 1). Stimulation of these alpha-adrenergic receptors results in smooth muscle contraction and increased outlet resistance, whereas alpha-adrenergic blockade results in smooth muscle relaxation and decreased bladder outlet resistance. Limitations of early alpha blockers included their side-effect profile with hypotension and dizziness. With later development of "selective" alpha blockers in the 1980s that targeted α-1 receptors rather than both α-1 and α-2 adrenergic receptors, these side effects were largely reduced.

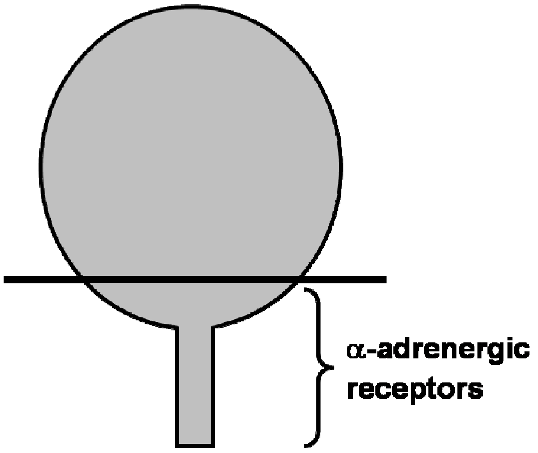

**FIGURE 1**. Location of alpha-adrenergic receptors in the LUT.

## ALPHA BLOCKER STUDIES IN PEDIATRIC LUT DYSFUNCTION

Today, alpha blockers are routinely used to facilitate bladder emptying in the adult population, particularly in adult males with benign prostatic hypertrophy[4]. We used this same treatment approach to report the first description of the efficacy of selective alpha blocker therapy to facilitate emptying in children with dysfunctional voiding[5]. In this pilot report, there was an 82% improvement in the measured parameters of 17 patients treated with alpha blocker therapy. Refractory response occurred in three of the four patients with Hinman syndrome (non-neurogenic, neurogenic bladder). Equally important, this pilot report demonstrated the safety profile of selective alpha blocker therapy in children; only one child reported postural hypotension that resolved by decreasing the alpha blocker dosage.

There have been several reports of selective alpha blocker therapy in the treatment of LUT dysfunction that have followed our initial report a decade ago.

I would emphasize that the historical timeline of these previously published alpha blocker reports in children limits the application of the current ICCS terminology with stringent uroflow criteria so as to be properly termed dysfunctional voiding. In fact, this was the impetus for the ICCS terminology paper; to emphasize uniformity and consistency to allow critical comparison of studies. Nevertheless, in a follow-up of our initial study, we continued to see improvement in multiple LUT symptoms and PVRs in 55 children treated with alpha blocker therapy for dysfunctional voiding[6]. Donohoe and associates reported the efficacy of alpha blocker therapy in 34 children with primary bladder neck dysfunction by demonstrating an improvement in the urine flow rate, reduction in PVR, and a decrease in the electromyography (EMG) “lag” time[7]. Yucel and colleagues compared alpha blocker therapy and biofeedback (pelvic floor urotherapy) to determine any difference in efficacy of these treatment modalities for dysfunctional voiding[8]. Both treatment modalities, alpha blocker therapy and biofeedback, showed significant improvement in the urine flow rate and the PVR. There was no difference in these improved voiding parameters between the two therapies, although there was a significant higher parental satisfaction in the alpha blocker therapy group compared to the biofeedback group. Limitations of this study and the earlier studies, however, include nonrandomization, patient heterogeneity, sample sizes, selection biases, and/or lack of a validated symptom score.

Kramer and colleagues conducted the first randomized, placebo-controlled study of selective alpha blocker therapy in children with dysfunctional voiding[9]. Approximately 40 children were randomized to either placebo vs. 0.5 mg of doxazosin. Unlike previous studies, they found no significant difference in the PVR and uroflow measurements between the treatment groups. There was a trend toward a significant improvement in urinary incontinence and the dysfunctional voiding symptom score in the alpha blocker group compared to the placebo group. Similar to the study by Yucel et al., there was a significant difference in parental satisfaction with a higher parental satisfaction in the children treated with alpha blockers compared to placebo. Although this study is the only placebo-controlled, randomized clinical trial (RCT) in children to use alpha blockers, the conclusions are significantly limited and should be interpreted accordingly, as with all of the published studies using alpha blockers in children with LUT dysfunction. In this study, there was no power analysis performed that typifies placebo-controlled RCTs and, therefore, it is limited by a potential inadequate sample size. Another significant limitation is the low, fixed doxazosin dosage employed by the investigators. In comparison to the previous alpha blocker studies that report efficacy in children with LUT dysfunction using dosages ranging from 0.5 to 8.0 mg[5,6,7,8,10], the Kramer study employed a substantially lower dosage (0.5 mg) and there was no consideration regarding the dose response of the subjects. Finally, the study was limited by usage of a nonvalidated symptom score tool to report their outcome variables.

### Safety Profile of Alpha Blockers in Children

Many medications used for LUT dysfunction, particularly the anticholinergics, are associated with dose-dependent side effects and, thus far, we have observed minimal side effects in children taking selective alpha blockers for dysfunctional voiding. The safety profile of the selective alpha blocker tamsulosin was recently examined in children treated for dysfunctional voiding[11]. In this study, there was no clinically significant effect on blood pressure, while tamsulosin proved to be a safe and effective treatment option for LUT dysfunction in a select pediatric population.

## CONCLUSION

In summary, alpha blocker therapy appears to be a safe treatment for dysfunctional voiding in children, and published reports are encouraging that alpha blockers improve emptying in children with LUT dysfunction. It should be noted that prescribing alpha blocker therapy to facilitate LUT emptying in children is off-label and there is a need for more substantive trials requiring industry and government grant support. Current limitations of trial design for patients with LUT dysfunction include the heterogeneity of these patients, the symptom-based components of their dysfunctional voiding, and the lack of validated study tools to measure outcomes. At present, alpha blocker therapy is an ancillary treatment for children with dysfunctional voiding and may be considered as an alternative to clean intermittent catheterization, biofeedback, and when other treatments have failed. Additionally, alpha blocker therapy may be used to facilitate behavioral modification in children with dysfunctional voiding.
